# Intrinsic Photosensitivity of the Vulnerable Seagrass *Phyllospadix iwatensis*: Photosystem II Oxygen-Evolving Complex Is Prone to Photoinactivation

**DOI:** 10.3389/fpls.2022.792059

**Published:** 2022-02-25

**Authors:** Mengxin Wang, Wei Zhao, Mingyu Ma, Di Zhang, Yun Wen, Mingyu Zhong, Chengying Luo, Zimin Hu, Quansheng Zhang

**Affiliations:** Ocean School, Yantai University, Yantai, China

**Keywords:** degeneration, oxygen-evolving complex, *Phyllospadix iwatensis*, photoinactivation, seagrass

## Abstract

*Phyllospadix iwatensis*, a foundation species of the angiosperm-dominated marine blue carbon ecosystems, has been recognized to be a vulnerable seagrass. Its degradation has previously been reported to be associated with environmental changes and human activities, while there has been a limited number of studies on its inherent characteristics. In this study, both the physiological and molecular biological data indicated that the oxygen-evolving complex (OEC) of *P. iwatensis* is prone to photoinactivation, which exhibits the light-dependent trait. When exposed to laboratory light intensities similar to typical midday conditions, <10% of the OEC was photoinactivated, and the remaining active OEC was sufficient to maintain normal photosynthetic activity. Moreover, the photoinactivated OEC could fully recover within the same day. However, under harsh light conditions, e.g., light intensities that simulate cloudless sunny neap tide days and continual sunny days, the OEC suffered irreversible photoinactivation, which subsequently resulted in damage to the photosystem II reaction centers and a reduction in the rate of O_2_ evolution. Furthermore, *in situ* measurements on a cloudless sunny neap tide day revealed both poor resilience and irreversible photoinactivation of the OEC. Based on these findings, we postulated that the OEC dysfunction induced by ambient harsh light conditions could be an important inherent reason for the degradation of *P. iwatensis.*

## Introduction

The seagrass *Phyllospadix iwatensis* (Zosteraceae), characterized by a well-developed root system and reddish-brown hairy fibers on the rhizomes, is naturally distributed in the rocky intertidal zone of the Northern Hemisphere (Cao et al., 2015; Li et al., 2020). As an important constituent species of angiosperm-dominated marine blue carbon ecosystems, *P. iwatensis* can form vast “underwater meadows,” which not only have an enormous ability to store carbon (Mcleod et al., 2011; Fourqurean et al., 2012) but also provide important habitat, spawning grounds, and food sources for various organisms in coastal areas (Cullen-Unsworth et al., 2018; Costa et al., 2020; Jiang et al., 2020; Rodriguez and Heck, 2020).

However, increasing amounts of research suggest that seagrasses are currently experiencing a global decline (Orth et al., 2006). *P. iwatensis* was assigned to the “vulnerable” category of the International Union for the Conservation of Nature red list in 2011 (Short et al., 2011). In China, the seaweed house, whose roof is primarily built by *P. iwatensis*, is a provincial intangible cultural heritage in Rongcheng, Shandong Province, with a history of >400 years (Yajing, 2021). However, seagrass beds in Shandong Province have shrunk by 90% during the past 20 years. At this point in time, *P. iwatensis* is scattered only in this coastal area to the extent that it is unable to meet the needs of housing restoration (Zheng et al., 2013).

Recently, with the extensive research on the degradation of seagrasses, most investigators believe that global climate change and human activities underlie the demise of their populations (Short and Wyllie-Echeverria, 1996; Short and Neckles, 1999). Since they inhabit shallow seas, seagrasses are exceptionally sensitive to changes in temperature and light. Therefore, an increase in the temperature of seawater owing to global climate change is an important cause of their degradation that cannot be ignored (Valle et al., 2014; Repolho et al., 2017; Duarte et al., 2018; Nguyen et al., 2021). Furthermore, eutrophication caused by human activities will induce the development of additional phytoplankton blooms, which can inhibit the photosynthesis performance of seagrass by reducing the light available (Hauxwell et al., 2003; Burkholder et al., 2007). Moreover, in many coastal areas, the fragmentation of habitat, pollution, overfishing, and biological invasion that can be caused directly by human activities also seriously threaten the survival of seagrasses (Short and Wyllie-Echeverria, 1996; Herrera-Silveira et al., 2010; Hall-Spencer and Harvey, 2019). Although the current causes of degradation have been studied extensively, less attention has been paid to the intrinsic vulnerability of seagrasses.

Oxidation of water molecules to electrons and molecular oxygen occurs in the oxygen-evolving complex (OEC) of photosystem II (PSII) (Cady et al., 2008; Gupta, 2020). The OEC is composed of manganese (Mn) ions, calcium (Ca) ions, peripheral proteins, and cofactors, and damage to each of these components results in the inactivation of the OEC (Nishimura et al., 2016). When the OEC is impaired, the lifetime of P680^+^ is prolonged, since the OEC cannot transfer electrons to the PSII reaction center quickly enough. P680^+^ is a strong oxidant that can oxidatively destroy the D1 protein in the PSII reaction center and then damage pigments such as carotenoids and chlorophyll (Tyystjärvi, 2008). We have recently observed that the OEC of the *Zostera marina* is preferentially inactivated under visible light (Tan et al., 2020; Zhao et al., 2021). Based on this, we chose to study the photosynthetic regulatory mechanisms induced by OEC photoinactivation under controlled laboratory conditions (Tan et al., 2020; Zhao et al., 2021). However, whether the dysfunction in OEC owing to photoinactivation can cause a decline in the productivity of seagrass, and even worse, in the degradation of population, is an extremely interesting topic that merits more attention.

In this article, the vulnerable seagrass *P. iwatensis* was utilized as the research object, and the OEC of *P. iwatensis* was confirmed to be prone to photoinactivation by the use of a mutually corroborative strategy of physiology and molecular biology. Furthermore, based on the fact that the photoinactivation of OEC inhibits its photosynthetic performance and even leads to irreversible damage in the photosynthetic apparatus, we suggest that the photoinactivation of OEC may be an important inherent reason for the degradation of *P. iwatensis.*

## Materials and Methods

### Plant Materials

**FIGURE 1:**
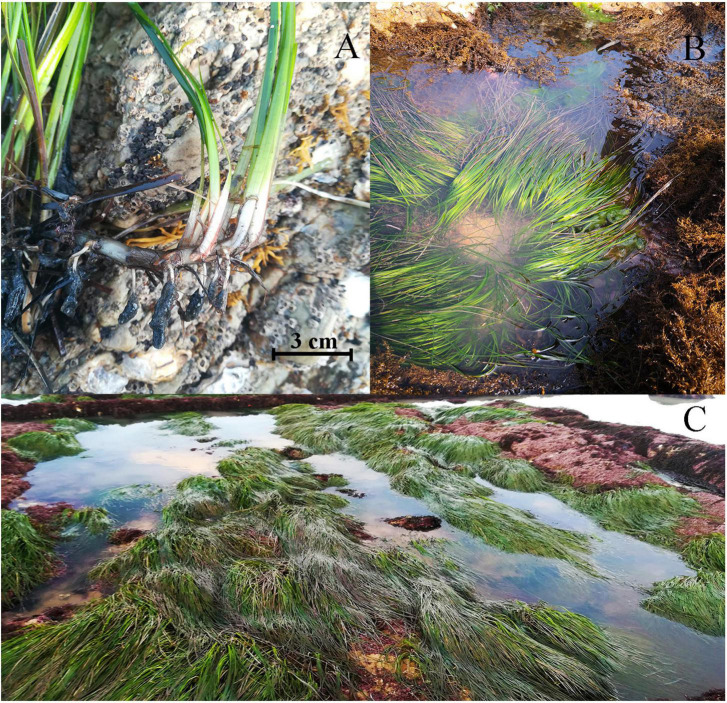
*Phyllospadix iwatensis*, characterized by reddish-brown hairy fibers at the base of the plant and a well-developed root system **(A)**, is naturally distributed in the rocky intertidal zone and can form vast “underwater meadows” **(B,C)**.

Healthy specimens of *P. iwatensis*, characterized by intact rhizome systems and fresh leaves that lacked mechanical injury (Figure 1), were collected from the rocky intertidal zone of Rongcheng (37° 16′N, 122° 41′E), Weihai, Shandong Province, China, throughout May 2021. The samples were precultured in aquaria with filtered seawater for 3 days under conditions of 15°C and a 10:14 h light: dark cycle with a minimum saturating irradiance of 40 μmol photons/m^2^ s.

### Experimental Treatments

Precultured *P. iwatensis* samples were acclimatized in the dark overnight prior to experimentation. To evaluate the dynamics of the OEC activity, dark-adapted samples were exposed to a light intensity of 400 μmol photons/m^2^ s, which was the usual underwater light conditions measured in *P. iwatensis* habitats during midday. The samples were exposed for 6 h, with measurements taken every 1 h during this period. Furthermore, to investigate the ability of OEC to recover, two light environments were simulated to treat the samples. Pretreated samples were exposed to 400 μmol photons/m^2^ s for 3 h to simulate the high light environment at midday under usual conditions. Following 1, 2, and 3 h of light exposure, a portion of the samples was taken for recovery under dark conditions in 15°C seawater. Measurements were taken every 1 and 3 h during exposure to light and recovery, respectively. In addition, based on the light environments of cloudless sunny neap tide days and continual sunny days, the times of exposure were extended to 6 h per day for 3 continuous days, which was established as the harsh light stress. Following light exposure, samples were recovered in the dark at 15°C. Measurements were taken at the beginning and end of each light exposure. All the experiments were conducted in aquaria. The light source was provided by an LED lamp with a color temperature range of 6,000 K (LI-COR Inc., Lincoln, NE, United States). The leaf segments were randomly collected from 2 cm above the leaf sheath before the parameters were measured, with a portion of the samples used for fluorescence measurements after 15 min of dark adaptation, while the other portions were immediately frozen in liquid nitrogen for subsequent Western blot analyses. The subscript “control” indicated that the parameter was derived from dark-adapted samples under controlled laboratory conditions. The subscript “treatment” indicated that the parameter was derived from samples treated with light stress in the laboratory. Each measurement was conducted in triplicate.

### Field *in situ* Measurements

To examine the *in situ* inactivation of the OEC, *in situ* measurements were conducted on a cloudless sunny neap tide day in May 2021 at the intertidal zone of a rocky shore with high seawater transparency in North Rongcheng (37°16’N, 122°41’E). Apparently healthy *P. iwatensis* plants were collected by diving every 2 h from 6:00 to 18:00 and at 6:00 and 8:00 on the following morning. Leaf segments that had been collected from ∼2 cm above the leaf sheath were partially used for chlorophyll fluorescence measurements, while the remainder were immediately frozen in liquid nitrogen for subsequent Western blot analyses. The light intensity of *P. iwatensis* habitats was measured using QSPL2100 (Biospherical Instruments Inc., San Diego, CA, United States). The subscript “control” indicated that the parameter was derived from samples collected at 6:00 on the first day of measurement under natural conditions in the field. The subscript “treatment” indicated that the parameter was derived from samples at different time points under natural conditions. Each measurement was conducted in triplicate.

### Chlorophyll a Fluorescence Measurements

To analyze the photosynthetic physiological parameters, M-PEA-2 (Hansatech, Norfolk, United Kingdom) was used to monitor the rapid chlorophyll fluorescence induction kinetic curve (OJIP curve). The OJIP curve was induced by 5,000 μmol photons/m^2^ s red light at a measurement time of 2 s. Chlorophyll fluorescence parameters were calculated as previously described (Strasser et al., 2010; Guo et al., 2020). The normalized OJIP fluorescence rise kinetics could be calculated using the formula Δ*Vt* = Δ[(*F*_*t*_ *– F_*O*_*)/(*F*_*m*_ *– F_*O*_*)]; *F*_*v*_/*F*_*m*_ = (*F*_*m*_ *– F_*O*_*)/*F*_*m*_, which represented the maximal quantum yield of the PSII; *W*_*K*_ = (*F*_*K*_ *– F*_*O*_)/(*F*_*J*_ *– F_*O*_*) and Δ*W*_*K*_ = [(*F*_*K*_ *– F*_*O*_)/(*F*_*J*_ *– F*_*O*_)]_treatment_ – [(*F*_*K*_ *– F*_*O*_)/(*F*_*J*_ *– F*_*O*_)]_control_, which reflected the extent of damage on the donor side of PSII; the active fraction of OEC centers, characterized by OEC_centers_, could be calculated as OEC_centers_ = [1 – (*V*_*K*_/*V*_*J*_)]_treatment_/[1 – (*V*_*K*_/*V*_*J*_)]_control_. A G-band observed between the I-step and P-step was supposed to relate to the redox state of the end PSI acceptor pool (Zagorchev et al., 2021).

### Western Blotting Analysis

The levels of expression of the OEC peripheral proteins PsbO, PsbP, PsbQ, and PSII core protein D1 were used to verify the activities of the OEC and PSII reaction center. The chloroplasts from leaves were separated using a Plants Leaf Chloroplast Rude Divide Kit (GenMED Scientifics Inc., Arlington, MA, United States). The chlorophyll content of the separated chloroplasts was measured as described earlier (Porra et al., 1989). To compare quantitative differences, serial dilutions (1.25, 2.5, and 5 μg of chlorophyll corresponding to 25, 50, and 100% of the control samples) of the control samples were loaded onto the first three lanes of a 12% SDS-triglycine-PAGE gel, followed by loading equal contents of chlorophyll (5 μg) of the solubilized materials of treated samples onto the same gel for separation. They were then transferred to PVDF membranes (0.22 μm; Sartorius Stedim, Göttingen, Germany). Western blot assays with antibodies against PsbO, PsbP, PsbQ, and D1 (Agrisera, Vännäa, Sweden) were performed as described earlier (Fristedt et al., 2009). RuBisCo large subunits (RbcL) with the same loading volume (5 μg of chlorophyll) as the proteins above were used as equal loading controls. The chemiluminescent bands were quantified on a Gel Doc XR + system (Bio-Rad, Hercules, CA, United States) using Image Lab software (Bio-Rad). The total sample density in each case was normalized based on the RbcL density. Each measurement was conducted in triplicate.

### Oxygen Evolution Rate Measurements

The rate of photosynthetic O_2_ evolution was measured to evaluate the overall photosynthetic performance. This rate was determined using a liquid-phase oxygen electrode system (Chlorolab2 + ; Hansatech, Norfolk, United Kingdom) at 15°C. Leaf fragments (∼25 mg) were placed in the reaction chamber with 2 ml of seawater. A white light of 40 μmol photons/m^2^ s was used to measure the evolution of O_2_. The net photosynthetic rate (Pn) of the leaves was measured within 3 min of light irradiation, and the respiration rate (R) was measured within 2 min of the dark state. The rate of O_2_ evolution (P) was calculated as *P* = *R* + *Pn* and expressed as nmol O_2_/min g fresh mass. Each measurement was conducted in triplicate.

### Data Analysis

A statistical analysis of the collected parameters was performed using a one-way ANOVA in SPSS 22.0 (IBM Inc., Armonk, NY, United States). *Post hoc* comparisons were made using Tukey’s trend test. Statistical significance was assessed at a threshold of *p* < 0.05.

## Results

### Photoinactivation of Oxygen-Evolving Complex

**FIGURE 2:**
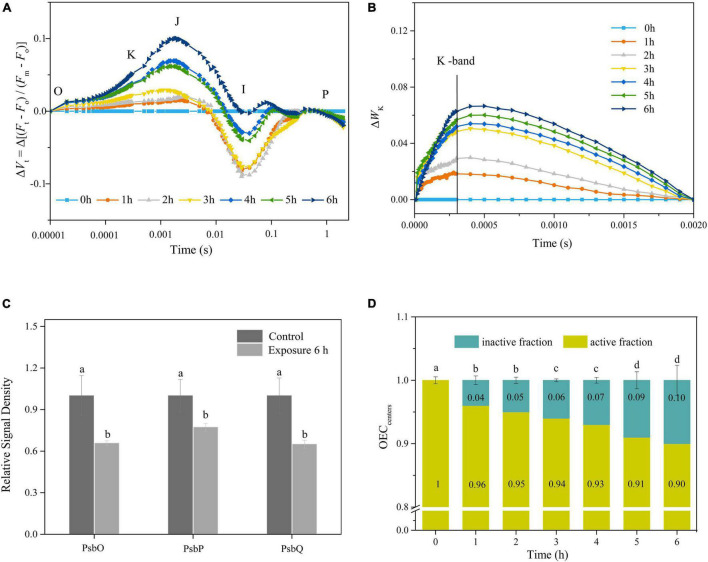
**(A,B)** Time courses of normalized chlorophyll a fluorescence intensity of Δ*V*_*t*_ and Δ*W*_*K*_ curves in response to HL (400 μmol photons/m^2^ s) exposure. Each curve represents the average of three replicates. **(C)** The relative expression levels of OEC peripheral proteins after 6 h HL exposure. The means ± SD were calculated from three independent samples. Different letters indicate a significant difference (Tukey’s tests, *p* < 0.05). **(D)** Variations in OEC_centers_ in response to HL exposure. The means ± SD were calculated from three independent samples. Different letters indicate a significant difference (Tukey’s tests, *p* < 0.05).

The Δ*Vt* curves of fluorescence rise kinetics normalized by OJIP changed with an increase in the time of exposure, suggesting that the performance of PSII was significantly affected by the duration of exposure (Figure 2A). The intensity of K-band at 0.3 ms in the curve of Δ*W*_*k*_ increased gradually with the duration of exposure, indicating that the OEC was continuously inactivated (Figure 2B). Furthermore, the activity of OEC, characterized by the peripheral protective protein contents of PsbO, PsbP, and PsbQ, decreased significantly during light exposure (Figure 2C and Supplementary Figure 1). Indeed, high light (HL) induced the partial inactivation of active OEC, as demonstrated by the variation in the physiological parameter OEC_centers_ (Figure 2D). The percentage of OEC inactivation continuously increased with the duration of exposure, with 6 h of exposure resulting in about 10% of inactivation (Figure 2D).

### Reversible Photoinactivation of Oxygen-Evolving Complex

**FIGURE 3:**
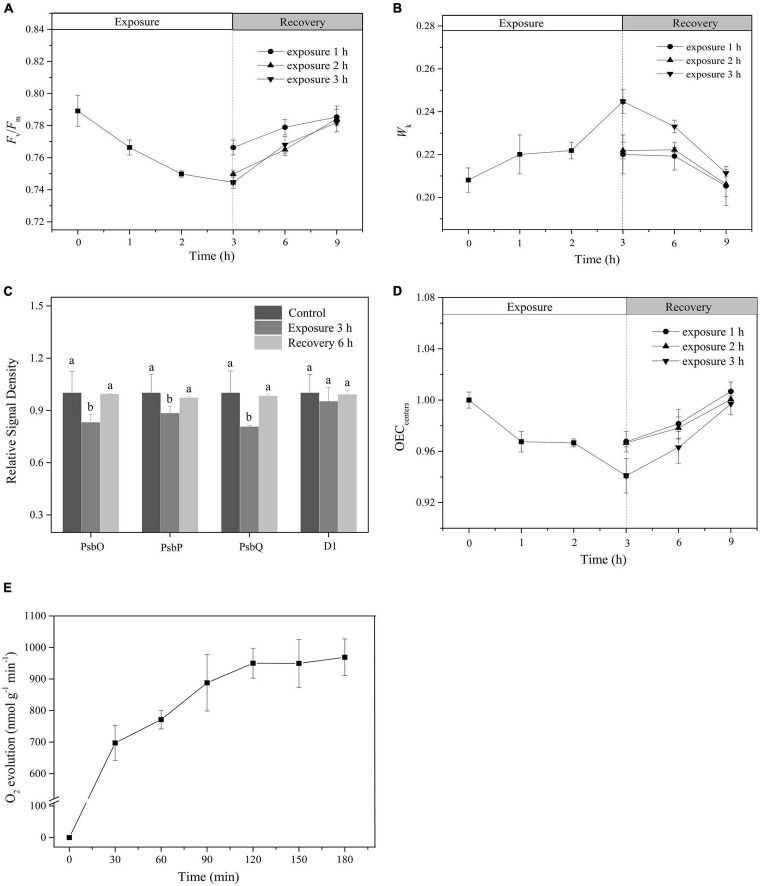
The recovery of photoinactivated OEC. **(A,B)** Time course of changes in *F*_*v*_/*F*_*m*_ and *W*_*K*_. White and light gray rectangles represent HL (400 μmol photons/m^2^ s) exposure and dark recovery period, respectively. Circle, triangle, and inverted triangle represent the different recovery start times: after HL exposure for 1, 2, and 3 h, respectively. **(C)** The relative expression levels of OEC peripheral proteins and PSII core D1 protein after 3 h HL exposure and subsequent 6 h recovery. The means ± SD were calculated from three independent samples. Different letters indicate a significant difference (Tukey’s tests, *p* < 0.05). **(D)** Time course of changes in OEC_centers_. White and light gray rectangles represent HL exposure and dark recovery period, respectively. Circle, triangle, and inverted triangle represent the different recovery start time: after HL exposure for 1, 2, and 3 h, respectively. **(E)** Changes in O_2_ evolution rate. The means ± SD were calculated from three independent samples.

Following a single HL exposure for 3 h, both the *F*_*v*_/*F*_*m*_ and *W*_*k*_ gradually recovered during darkness (Figures 3A,B). Furthermore, the Western blot showed that the protein contents of PsbO, PsbP, and PsbQ increased during the recovery period (Figure 3C and Supplementary Figure 1). All these parameters reached their initial levels after 6 h of recovery, which was also confirmed by the full recovery of OEC_centers_ (Tukey’s test, *p* = 0.171, *p* = 0.13, *p* = 0.055, *p* = 0.073, *p* = 0.068, and *p* = 0.54, respectively; Figures 3A–D). No net loss of the D1 protein was observed during illumination (Tukey’s test, *p* = 0.86; Figure 3C and Supplementary Figure 1), indicating that the PSII reaction centers did not suffer net damage.

To further verify the effect of light exposure on the photosynthetic performance of *P. iwatensis*, the rate of O_2_ evolution was measured. Within a single 3-h period of exposure to HL, the rate of O_2_ evolution gradually increased, exhibiting a typical photoinduction process (Figure 3E), which represents a normal photosynthetic performance.

### Irreversible Photoinactivation of Oxygen-Evolving Complex

**FIGURE 4:**
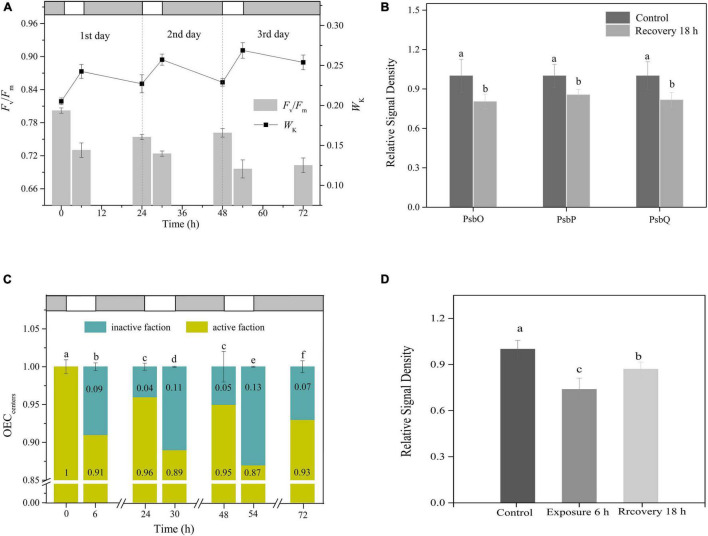
Irreversible photoinactivation of OEC. **(A)** Time courses of changes in *F*_*v*_/*F*_*m*_ and *W*_*K*_ in response to 6 h of HL (400 μmol photons/m^2^ s) exposure per day for 3 continuous days. White and light gray rectangles represent HL exposure and dark recovery period, respectively. **(B)** The relative expression levels of OEC peripheral proteins after recovery from 6 h HL exposure. The means ± SD were calculated from three independent samples. Different letters indicate a significant difference (Tukey’s tests, *p* < 0.05). **(C)** The changes in OEC_centers_. White and light gray rectangles represent HL exposure and dark recovery period, respectively. Different letters indicate a significant difference (Tukey’s tests, *p* < 0.05). **(D)** The relative expression levels of PSII core protein D1 in response to 6 h HL and the subsequent recovery for 18 h. The means ± SD were calculated from three independent samples. Different letters indicate a significant difference (Tukey’s tests, *p* < 0.05).

Following a single 6-h exposure, *F*_*v*_/*F*_*m*_ and *W*_*k*_ failed to reach their initial levels until the following day (Tukey’s test, *p* < 0.05, *p* < 0.05, respectively; Figure 4A). This was also true for the changes in both the OEC peripheral protein contents and OEC_centers_ (Tukey’s test, *p* < 0.05, for both OEC peripheral proteins and OEC_centers_; Figures 4B,C and Supplementary Figure 1). These observations indicated the occurrence of irreversible photoinactivation of OEC. Furthermore, irreversible oxidative damage to the PSII reaction centers was observed, as indicated by the D1 protein, which suffered a net loss during illumination and did not return to its original state by the morning of the next day (Tukey’s test, *p* < 0.05; Figure 4D and Supplementary Figure 1).

During 6 h of HL exposure per day for 3 continuous days, *F*_*v*_/*F*_*m*_ and *W*_*k*_ exhibited rhythmic changes of the light-dark cycle, with gradual decreases and increases, respectively, during the day and incomplete recovery at night, which made *F*_*v*_/*F*_*m*_ and *W*_*k*_ keep decreasing and increasing as the days progressed (Figure 4A). Moreover, aside from the decrease in OEC_centers_ with the extended days of exposure, the proportion of OEC that did not regain activity after dark adaptation overnight also increased daily (Figure 4C). These results indicated the occurrence of continuous irreversible photoinactivation of OEC.

**FIGURE 5:**
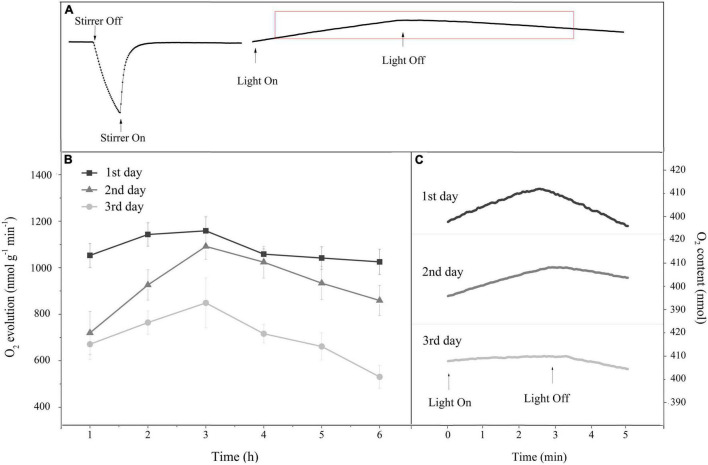
**(A)** A typical oxygen content change curve in the reaction chamber of the Liquid-Phase Oxygen Electrode System for monitoring the O_2_ evolution rate. **(B)** Time course of the O_2_ evolution rate in response to 6 h of HL (400 μmol photons/m^2^ s) exposure per day for 3 continuous days. Square, triangle, and circle represent the continuous exposure for 1st, 2nd, and 3rd day, respectively. The means ± SD were calculated from three independent samples. **(C)** Changes in the oxygen content curve during the O_2_ evolution rate monitoring after 6 h of continuous HL exposure per day. The rising slope of the curve under light represents the net photosynthetic rate, and the falling slope of the curve in darkness represents the respiration rate. Each curve is based on the average of three replicates.

The complete measurement process of the O_2_ evolution rate is shown in Figure 5A. When light exposure exceeded 3 h, the rate of O_2_ evolution decreased gradually, indicating that the photosynthetic performance was inhibited following long light exposure (Figure 5B). Additionally, under daily light stress, both the net photosynthetic rate in the light and the respiration rate in the dark decreased daily (Figure 5C). The O_2_ evolution rate calculated from them exhibited a similar trend (Figure 5B).

### *In situ* Measurements of Oxygen-Evolving Complex Activity

**FIGURE 6:**
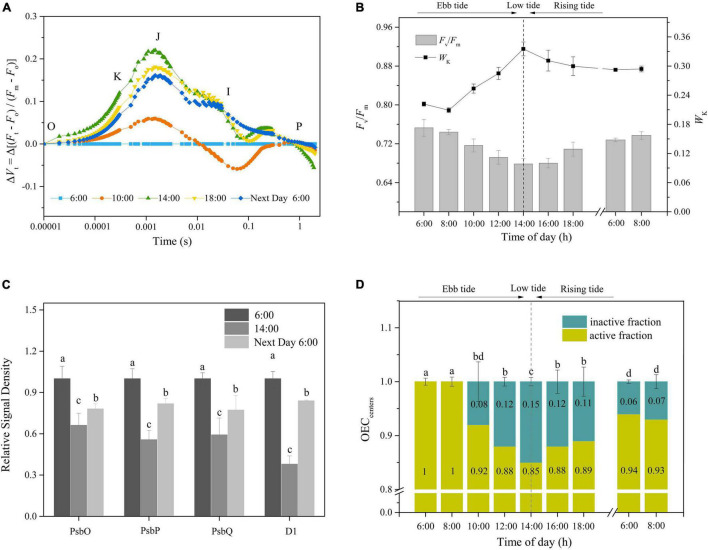
The *in situ* occurrences of OEC photoinactivation. **(A,B)** Changes in the Δ*V*_*t*_ curves, *F*_*v*_/*F*_*m*_, and *W*_*K*_ in response to the tidal cycle. **(C)** The relative protein expression levels of PsbO, PsbP, PsbQ, and D1 in the early morning, lowest tide, and after dark recovery overnight. The means ± SD were calculated from three independent samples. Different letters indicate a significant difference (Tukey’s tests, *p* < 0.05). **(D)** Time courses of OEC_centers_ in response to the tidal cycle. Different letters indicate a significant difference (Tukey’s tests, *p* < 0.05). The photosynthetic photon flux density (PPFD) at 8:00, 10:00, 12:00, 14:00, and 16:00 was around 100, 250, 350, 400, and 300 μmol photons/m^2^ s, respectively. The means ± SD were calculated from three independent samples.

To gain insight into the OEC photoinactivation of *P. iwatensis* in its natural environment, the chlorophyll fluorescence and protein contents were measured *in situ* on seagrass beds. As shown in Figure 6A, the Δ*Vt* curves significantly fluctuated in a diurnal manner. In the morning, the decrease in *F*_*v*_/*F*_*m*_ was accompanied by an increase in *W*_*k*_, reaching their extreme values at 14:00. In the afternoon, *F*_*v*_/*F*_*m*_ and *W*_*k*_ gradually recovered as the light intensity diminished, but their full recoveries were not observed by the next morning (Tukey’s test, *p* < 0.05, *p* < 0.05, respectively; Figure 6B). Similarly, an incomplete recovery was also demonstrated in the OEC peripheral proteins and PSII core D1 protein contents (Tukey’s test, *p* < 0.05, for both OEC and D1 proteins; Figure 6C and Supplementary Figure 2). The OEC_centers_, as shown in Figure 6D, changed with the process of diurnal light fluctuations, with the highest rate of inactivation of approximately 15% at 14:00, which remained at 6% inactivation following an overnight recovery (Tukey’s test, *p* < 0.05). Those results confirmed that *P. iwatensis* suffers irreversible OEC photoinactivation in a natural harsh light environment.

## Discussion

The manganese mechanism that is derived from the light-induced inactivation of the Mn cluster is an important component of the hypothesis of photo-inhibition (Ohnishi et al., 2005). This hypothesis considers that the light absorption by the Mn cluster in PSII leads to the release of Mn and the inactivation of OEC. This initial event precedes the damage to the PSII reaction center by light absorption by chlorophyll. A seminal work in pumpkin (*Cucurbita pepo*) showed that the release of an Mn ion into the thylakoid lumen was the earliest detectable step of photo-inhibition and that the action spectrum of photo-inhibition resembled the absorption spectra of Mn (III) and Mn (IV) compounds (Hakala et al., 2005). Subsequently, Mn photo-inhibition was observed by Sarvikas et al. (2006) in *Arabidopsis*, Oguchi et al. (2009) in capsicum (*Capsicum annuum*), He et al. (2015) in barley (*Hordeum vulgare*), and Oguchi et al. (2011a) and Iermak et al. (2020) in spinach (*Spinacia oleracea*), among others. Moreover, Oguchi et al. (2009, 2011a,2011b) suggested that both the energy excess mechanism, which states that excess energy absorbed by chlorophyll leads to PSII photoinactivation, and the manganese mechanism, operate during the process of photo-inhibition, with the relative contribution of each mechanism depending on the plant species or growth conditions. All the studies described are fundamental for the corpus of manganese mechanism. In this mechanism, the OEC is inactivated by light absorbed by the Mn cluster, and a positive band in the OJIP curve at 0.3 ms, designated the K-step, may serve as a specific marker of OEC damage (Iermak et al., 2020).

In our study, both the data of relative variable fluorescence at the K point and abundance in the OEC peripheral stabilizing proteins PsbO, PsbP, and PsbQ confirmed the fact that the OEC of *P. iwatensis* was prone to photoinactivation, which was consistent with the characteristics of manganese mechanism. *P. iwatensis* primarily naturally inhabits intertidal areas where the light fluctuation depends on both daily variations and tidal changes. In our simulated experiments, the short light exposure (3 h) induced the increase in *W*_*k*_ and the decrease in OEC peripheral protein contents and OEC_centers_ (<10%), which recovered rapidly as samples were transferred to the dark, suggesting an occurrence of the reversible photoinactivation of OEC. Moreover, the data for D1 protein content and the evolution of photosynthetic O_2_ showed that photosynthesis was not significantly affected. As observed in *Z. marina*, when the OEC is partially inactivated, PSII-CEF and ascorbic acid (AsA) can supply electrons to the reaction centers, while the antioxidant system and controlled selective electron flow is not significantly activated, thereby allowing the photosynthetic system to efficiently use the limited electrons to maintain normal levels of carbon assimilation (Zhao et al., 2021). PSI-CEF operates efficiently to establish ΔpH, contributing to the maintenance of OEC stability (Tan et al., 2020). In contrast, after 6 h of light exposure, the *F*_*v*_/*F*_*m*_, *W*_*k*_, OEC peripheral protein contents, and the OEC_centers_ varied significantly and could not recover completely after spending overnight in the dark, indicating an occurrence of irreversible photoinactivation on both the OEC and PSII. Furthermore, the net loss of D1 protein and the reduced rate of O_2_ evolution further suggested that a net damage to the photosynthetic system occurred. In terms of the continuous daily exposure, both the chlorophyll fluorescence and protein data showed the accumulation of inactivated OEC. The gradual decrease in the rate of O_2_ evolution also indicated a continuous impairment of the photosynthetic performance. Based on these results, we postulated that the natural environments of cloudless sunny neap tide days and continuous sunny days under high seawater transparency could lead to the irreversible photoinactivation of OEC in *P. iwatensis.* To verify this hypothesis, the OEC activity of *P. iwatensis* was determined by *in situ* measurements on a cloudless sunny neap tide day. The lowest tide is at approximately midday, during neap tides, and therefore coincides with the most serious light exposure. As expected, both chlorophyll fluorescence and the level of expression of key proteins exhibited similar trends with the observations in the laboratory experiments, with the changes of *W*_*k*_, *F*_*v*_/*F*_*m*_, OEC peripheral proteins, and OEC_centers_ induced by light exposure had not fully recovered by the next morning, revealing the poor resilience of OEC. The 15 and 6% inactivation of OEC at the lowest tide and the following morning, respectively, indicated that the OEC had suffered irreversible damage under field environments. The irreversible photoinactivation of the OEC will undoubtedly affect the photosynthetic performance and may subsequently lead to plant mortality and population degradation. Therefore, we propose that the dysfunction of OEC induced by natural harsh light environments is probably an important inherent reason for the degradation of *P. iwatensis.*

The OEC of seagrasses prone to photoinactivation may be a result of the lack of protection by substances that function to shield the light owing to the absence of photoreceptors (Olsen et al., 2016; Ma et al., 2021). Additionally, chloroplasts are located on the external epidermis of leaves, which facilitate the transport and diffusion of inorganic carbon and also render the OEC prone to photoinactivation owing to the maximum light received by the chloroplasts (Beer et al., 2014).

## Conclusion

The OEC of *P. iwatensis* is prone to photoinactivation, and it occurs in natural environments. Generally, the photoinactivation of OEC in *P. iwatensis* can be fully recovered on the same day without affecting the photosynthetic performance. However, the resilience of OEC is poor under harsh light conditions. In this case, the reduced photosynthetic performance and even the damaged photosynthetic apparatus caused by the irreversible photoinactivation of OEC will threaten the survival of seagrasses. Therefore, we suggest that OEC dysfunction induced by harsh light conditions such as cloudless sunny neap tide days and continuous sunny days under high transparency seawater may trigger destructive chronic impacts on *P. iwatensis* that can result in its degradation.

## Data Availability Statement

The original contributions presented in the study are included in the article/Supplementary Material, further inquiries can be directed to the corresponding author.

## Author Contributions

QZ got the project funding. QZ, WZ, MM, MZ, CL, and ZH designed the experiment. MW, WZ, MM, and YW carried out the experiment and analyzed the data. MW wrote the first version of the manuscript. DZ and QZ made significant contributions to the manuscript and critically revised the different versions of the manuscript. All authors contributed to the article and approved the submitted version.

## Conflict of Interest

The authors declare that the research was conducted in the absence of any commercial or financial relationships that could be construed as a potential conflict of interest.

## Publisher’s Note

All claims expressed in this article are solely those of the authors and do not necessarily represent those of their affiliated organizations, or those of the publisher, the editors and the reviewers. Any product that may be evaluated in this article, or claim that may be made by its manufacturer, is not guaranteed or endorsed by the publisher.
