# Supplementary material for: Intrinsic Photosensitivity of the Vulnerable Seagrass Phyllospadix iwatensis: Photosystem II Oxygen-Evolving Complex Is Prone to Photoinactivation
Source: Front Plant Sci. 2022 Feb 25;13:792059. doi: 10.3389/fpls.2022.792059 (PMC8914196; doi:10.3389/fpls.2022.792059)
Supplement: Supplementary file 1 [file Data_Sheet_1.docx]

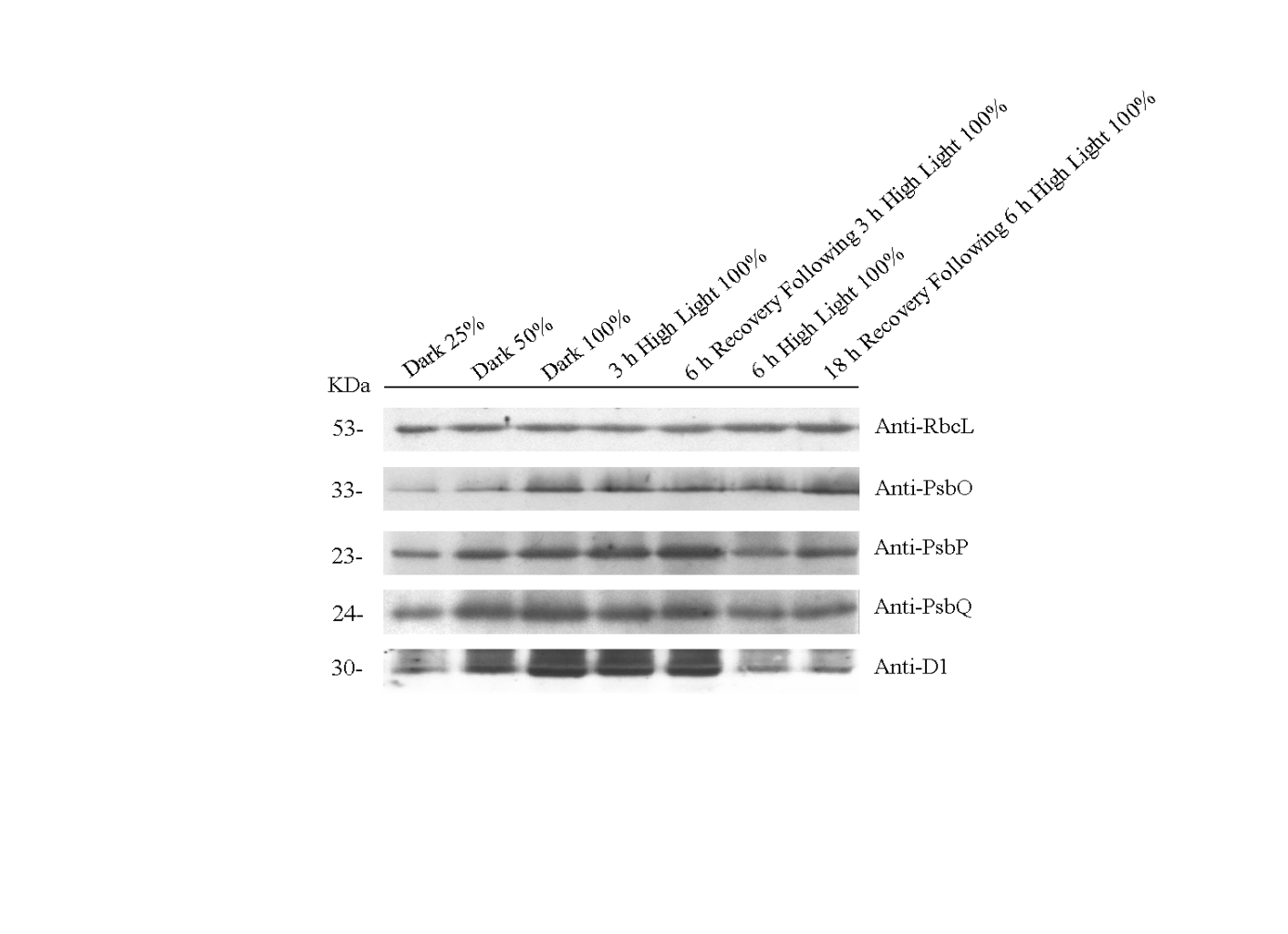


Figure S1. Expression levels of OEC peripheral protective proteins and PSII core D1 protein were analyzed by Western blot using specific antibodies under control condition, HL (400 μmol photons m^−2^ s^−1^) exposure and recovery.


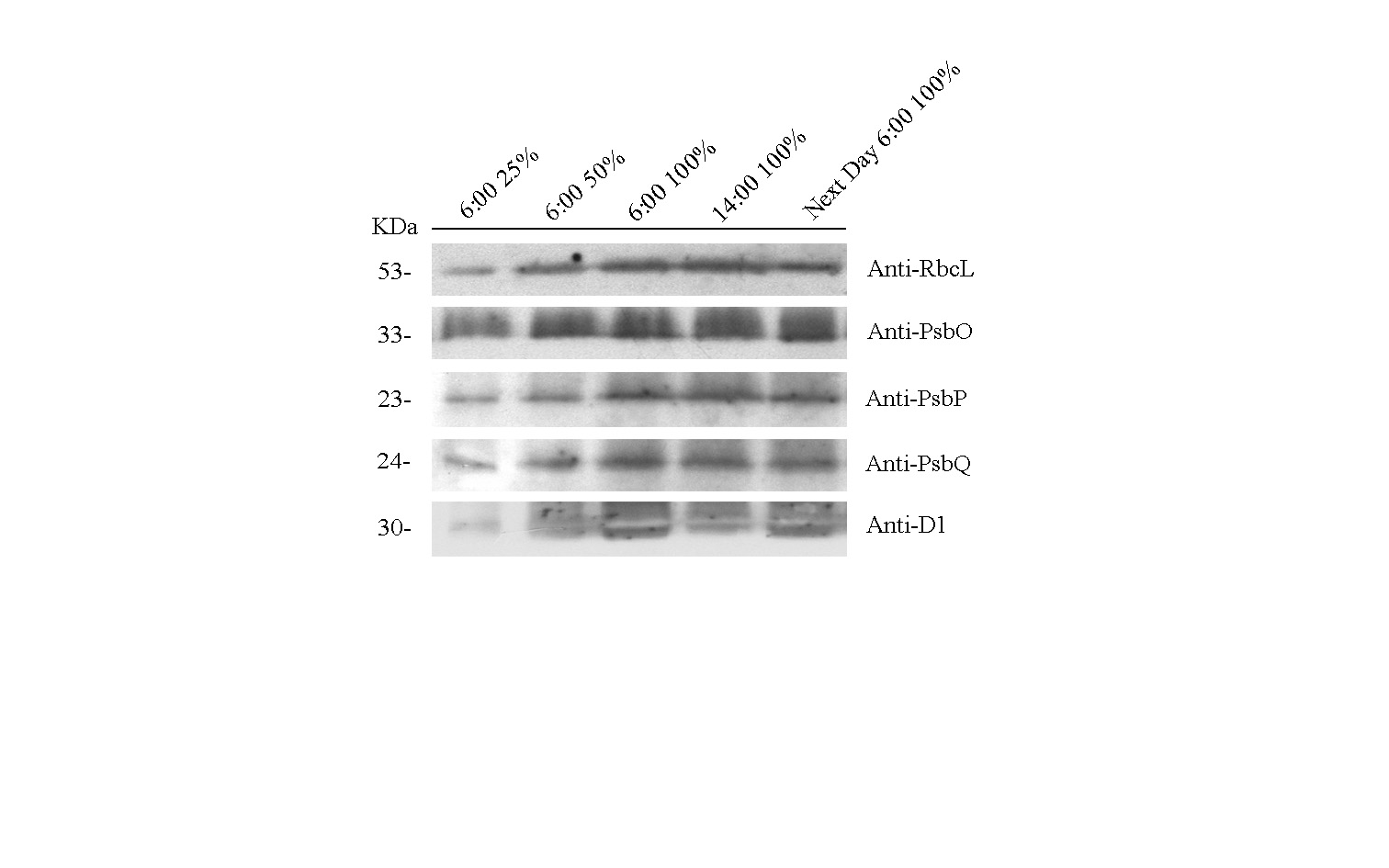


Figure S2. Expression levels of OEC peripheral protective proteins and PSII core D1 protein in the early morning, lowest tide and after dark recovery overnight were analyzed by Western blot using specific antibodies.
